# Serum *N*-glycome characterization and anti-carbohydrate antibody profiling in oral squamous cell carcinoma patients

**DOI:** 10.1371/journal.pone.0178927

**Published:** 2017-06-08

**Authors:** Shih-Yun Guu, Tsung-Hsien Lin, Su-Chieh Chang, Rei-Jing Wang, Ling-Yi Hung, Po-Jan Fang, Wei-Chien Tang, Peiwen Yu, Chuan-Fa Chang

**Affiliations:** 1 Department of Medical Laboratory Science and Biotechnology, College of Medicine, National Cheng Kung University, Tainan, Taiwan; 2 Institute of Basic Medical Sciences, College of Medicine, National Cheng Kung University, Tainan, Taiwan; 3 OBI Pharma, Inc., Taipei, Taiwan, R.O.C; 4 Center of Infectious Disease and Signaling Research, College of Medicine, National Cheng Kung University, Tainan, Taiwan; 5 Department of Medical Laboratory Science and Biotechnology, College of Health Sciences, Kaohsiung Medical University, Kaohsiung, Taiwan; Università degli Studi della Campania "Luigi Vanvitelli", ITALY

## Abstract

Glycosylation is a protein post translational modification which plays important role in protein function, stabilization, trafficking, and turnover. Alteration of protein glycosylation is a common phenomenon during tumor progression, migration, invasion, angiogenesis, as well as metastasis. Hence, aberrant glycan structures and the induced corresponding anti-carbohydrate antibodies are potential biomarkers for cancer diagnosis. In this study, serum *N*-glycomes and anti-carbohydrate antibodies from normal populations and oral squamous cell carcinoma (OSCC) patients were investigated. Total serum proteins were lyophilized and subjected to chemical reduction, alkylation and trypsin digestion. The *N*-glycans were released, purified, permethylated, and analyzed using MALDI-TOF-Mass spectrometry. In addition, the serum anti-carbohydrate antibody profiles were also investigated by carbohydrate microarray. We found that the relative abundances of seven *N*-glycans were decreased or increased in serum of OSCC with diagnostic accuracy greater than 75%. The relative abundances of total tri-antennary and tetra-antennary glycans with varying degrees of fucosylation and sialylation were also increased in serum *N*-glycomes of OSCC. In an independent validation group of forty-eight OCCC patients, most of the high-molecular weight serum *N*-glycans showed significantly high sensitivity and specificity according to the identified cutoff values. Furthermore, the serum levels of two IgM antibodies were elevated accompanied with the decreased levels of nine IgG antibodies in patient serum. Taken together, these serum *N*-glycans and antibodies identified in this study should be considered as the candidates of potential biomarkers for OSCC diagnosis.

## Introduction

Oral squamous cell carcinoma (OSCC) is the tumor that grows on the lips, tongue, floor of the oral cavity, hard and soft palate, sinuses, salivary glands, tonsils, pharynx and the peripheral tissue of the mouth. It is one of the most commonly diagnosed cancer in the world. In Taiwan, OSCC is the sixth highest incidence and the fifth highest mortality of the malignancy [1]. The relative prevalence of OSCC increases about 110% over the past 15 years because of the habit of smoking, drinking and betel quid chewing [2, 3]. In addition, the five-year-survival rate of OSCC is also unsatisfied in late stage (52.2% in stage III and 32.8% in stage IV) (Bureau of Health Promotion Department of Health, R.O.C., Taiwan). Therefore, the high prevalence and mortality rate of OSCC in Taiwan makes it important to investigate biomarkers for the surveillance of high-risk population to early intervention of OSCC.

The concept of alteration of glycosylation pattern in malignancy is first described by Meezan *et al*. and Wu *et al*. in 1969 who demonstrated that the content of neutral and amino sugars, especially sialic acid and *N*-acetylgalactosamine are markedly decreased in the membrane glycoproteins of the virus-transformed fibroblasts [4, 5]. Until now, more and more studies showed that aberrant glycosylation is implicated in different type of cancers and can be associated with the progression of malignancy by affecting the growth and proliferation, invasion, metastasis, angiogenesis and behavior to immunity of the tumor [6, 7]. The common alteration of glycosylation pattern found in cancer cells can be loss of expression or excessive expression of certain structures, truncated or altered branching patterns of certain glycans, and sometimes, the appearance of novel structures [8]. The alteration of glycosylation can be one of the hallmarks of cancer. Several carbohydrate-related tumor antigens such as sLe^x^, sLe^a^, sTn, TF, Le^y^, Globo H, PSA, GD2, GD3, fucosyl GM1 and GM2 have been demonstrated to be diagnostic or prognostic marker in cancer [9–11]. For instance, serum *N*-glycans are potential biomarker for cancer diagnosis or prognosis for ovarian cancer, prostate cancer, breast cancer, lung cancer and esophageal adenocarcinoma [12–16]. In addition, these carbohydrate-related tumor antigens are also studied for being the target of the new anti-cancer drugs or carbohydrate-based anticancer vaccines [17–22]. For example, anti-GD2 antibodies combined with cytokines and isotretinoin can significantly improve the event-free survival of neuroblastoma patients in a phase III clinical trial [23]. The GH (Globo H)-DT/C34 vaccine which can elicit antibodies against Globo H, stage-specific embryonic antigen 3 (SSEA3) and SSEA4 which are specifically observed in breast cancer cells and cancer stem cells is now in preclinical test and about to apply for the clinical trial in Taiwan [19]. Furthermore, the generated antibodies against carbohydrate tumor markers have also been applied for diagnosis of cancer malignancy and prognosis after treatments [24–28].

Although some aberrant glycosylation in serum or malignant tissues and some proteomic/genomic biomarkers in salivary have been reported to be associated with cellular invasion and the stage of OSCC [29–36], the lack of high sensitivity/specificity biomarkers for OSCC diagnosis is still a serious public health problem in many countries. In this study, we profiled serum *N*-glycomes and screened anti-carbohydrate antibodies from OSCC patient and normal volunteer by mass spectrometry and carbohydrate chip, respectively. Several *N*-glycans and antibodies with high diagnostic accuracy were identified and validated. Based on our findings, these serum *N*-glycans and antibodies should be considered as the candidates of potential biomarkers for OSCC diagnosis.

## Materials and methods

### Serum samples

This study was approved by National Cheng Kung University Hospital Institutional Review Board. The serum samples of OSCC patients (N = 65) were obtained from the tissue bank of National Cheng Kung University Hospital. The need for participant consent was waived by the ethics committee for serum sample form tissue bank. The venous blood from cancer-free healthy volunteers (N = 21, test group; N = 6, validation group) and OSCC patients (N = 48, validation group) were collected by BD Vacutainer^™^ without anticoagulant. The characteristics of the cancer-free volunteers and OSCC patients are listed in S1 and S2 Tables. All the participants in this study are adult. The information on the approved participant consent form was introduced verbally to every participant by medical technologist before blood drawing. The participants also signed on the approved participant consent form. The whole blood was allowed for clot formation at room temperature within 30 minutes. The whole blood was centrifuged at 3000 rpm (KUBOTA/KN70) at room temperature for 10 minutes and the upper serum layer was acquired. All serum samples were stored at -80°C until the process of glycomics analysis.

### *N*-glycan release and purification

The procedure of glycomics analysis was modified from literature [37]. A 20 μL of serum was lyophilized and dissolved in 1 mL of denaturing buffer (6 M guanidine hydrochloride in 1 mM CaCl_2_, 0.1 M Tri-HCl, pH = 8.6) and agitated at 4°C overnight. For reduction of the serum proteins, dithiothreitol (DTT) was added to obtain a final concentration of 20 mM and the sample was incubated at 37°C for 3 hours. For protein alkylation, iodoacetamide (IAA) was added to a final concentration of 50 mM and the sample was incubated in the dark at 37°C for 3 hours. Next, the sample was dialyzed against ddH_2_O at 4°C for 2 days with several times of change of ddH_2_O to remove the excess DTT and IAA. Then the sample was dried by spin-vacuum. The dried sample was resuspended in 200 μL of 50 mM NH_4_HCO_3_ solution (pH = 8.3) containing 2 mg of TPCK-treated trypsin and incubated at 37°C for at least 24 hours. The enzyme activity of trypsin was destroyed by heating the sample solution at 100°C for 10 minutes. Finally, the *N*-glycans were enzymatically released from the protein backbone by adding PNGase F to the sample solution to obtain a final concentration of 2.5 U/μL. The sample was incubated at 37°C for at least 18 hours. For purification of the released *N*-glycans, C_18_ Sep-Pak cartridge was used to exclude the deglycosylated peptides. The C_18_ Sep-Pak cartridge was first sequentially conditioned with 5ml of methanol, 5 mL of acetone and then 5 mL of 5% acetic acid. Then the fraction with released *N*-glycans was eluted by 6 mL of 5% acetic acid and the deglycosylated peptides were eluted by 6 mL of 60% 1-propanol. Both the eluted fractions were dried by spin-vacuum.

### Permethylation of *N*-glycans

The purified *N*-glycans were permethylated by NaOH/dimethyl sulfoxide slurry method [38]. The dried N-glycans were mixed with 100 μL of NaOH-dimethyl sulfoxide slurry and 100 μL of methyl iodide and then agitated vigorously at room temperature for 1 hour. Afterward, about 1.5 mL of ddH_2_O was added into the mixture to quench the reaction. To extract the permethylated N-glycans, 1 mL of chloroform was added and the chloroform phase was washed 10 times with 1.5 mL of ddH_2_O. Finally, the aqueous phase was discarded and the chloroform phase containing permethylated N-glycans was dried by a stream of nitrogen.

### MALDI-TOF MS analysis

First, the permethylated N-glycans were dissolved in 20 μL of acetonitrile and 1 μL of the sample was mixed with 1 μL of matrix buffer (methanol/1 mM sodium acetate (1:1, vol/vol) containing 10 mg of 2,5 dihydroxybenzoic acid (DHB) per mL). Then 2 μL of the sample-matrix mixture was spotted directly on the MALDI-TOF target plate and allowed to dry at room temperature. Each sample was spotted on the plate in duplicate. The MALDI-TOF-MS spectras of the samples were acquired by Bruker Autoflex III MALDI-TOF (Bruker) in the positive-ion mode with the m/z range spreading from 1500 to 5000. A total of 1000 laser shots were operated to each sample spot each time. Each sample spot was operated at least 5 times.

### Anti-carbohydrate antibody profiling

20- and 40-fold serial dilutions were prepared by adding 25 μL of serum to 475 μL of Sample diluent, and adding 250 μL of 20-fold diluted serum to 250 μL of Sample diluent. 620 μL of Wash buffer, 120 μL of Blocking buffer, and 120 μL of diluted secondary antibody, were added into “Wash”, “Blocking” and “Conjugate” hole on the chip, respectively. Then, 100 μL of diluted sample was added to “Serum” hole on the chip. The chip was subjected into Pumping machine to start the reaction. After the reaction finished, 120 μL of mixed Substrates was added into “Substrate” hole on the chip. The binding intensity was measured by CCD analyzer.

### Data evaluation

The acquired MALDI-TOF-MS spectras were exported to a text file listing m/z values and intensities by using flexAnalysis (version 3.3, Bruker), a software provided by Bruker. Then, the primary glycan structures of major signals showed in serum-derived *N*-glycans MALDI-TOF-MS spectra were predicted by GlycoWorkbench, a software published by EUROCarbDB [39, 40] and the MS spectra with annotated glycan structures was exported. Three MS spectras of each sample were chosen randomly to the next process of data analysis. The relative intensity of each proposed *N*-glycan structure was calculated by expressing the intensity of each glycan ion as percent of the total intensity of all glycan ions. After normalizing, the relative intensity of each glycan ion was further used for statistical analysis. The “diagnostic performance” of each glycan structure was first evaluated by performing a nonparametric Mann-Whitney test using GraphPad Prism (version 5.0, GraphPad Software, Inc). The glycomic data with statistical significance (*p*-value less than 0.05) were assessed by the receiver-operator characteristics (ROC) test using GraphPad Prism (version 5.0, GraphPad Software, Inc). The glycan structures with diagnostic potential (area under curve, AUC>0.7) were investigated. The diagnostic sensitivity, specificity and accuracy of these structures for detecting OSCC were evaluated by 2 way contingency table analysis and the diagnostic significances were analysis by fisher's exact test using GraphPad Prism (version 5.0, GraphPad Software, Inc). The optimal cutoff value was determined based on Youden’s index (J) [41]. The correlation between the identified glycan structure and the metastatic status of OSCC and anti-carbohydrate antibody levels between normal with OSCC patient were analyzed by nonparametric Mann-Whitney test by GraphPad Prism (version 5.0, GraphPad Software, Inc). The correlations between the identified glycan structure and the stage of OSCC were analyzed by nonparametric Kruskal Wallis test with Dunn's test as the posthoc comparisons by GraphPad Prism.

## Results

### MS analysis of *N*-glycans released from the serum glycoproteins

**Fig 1:**
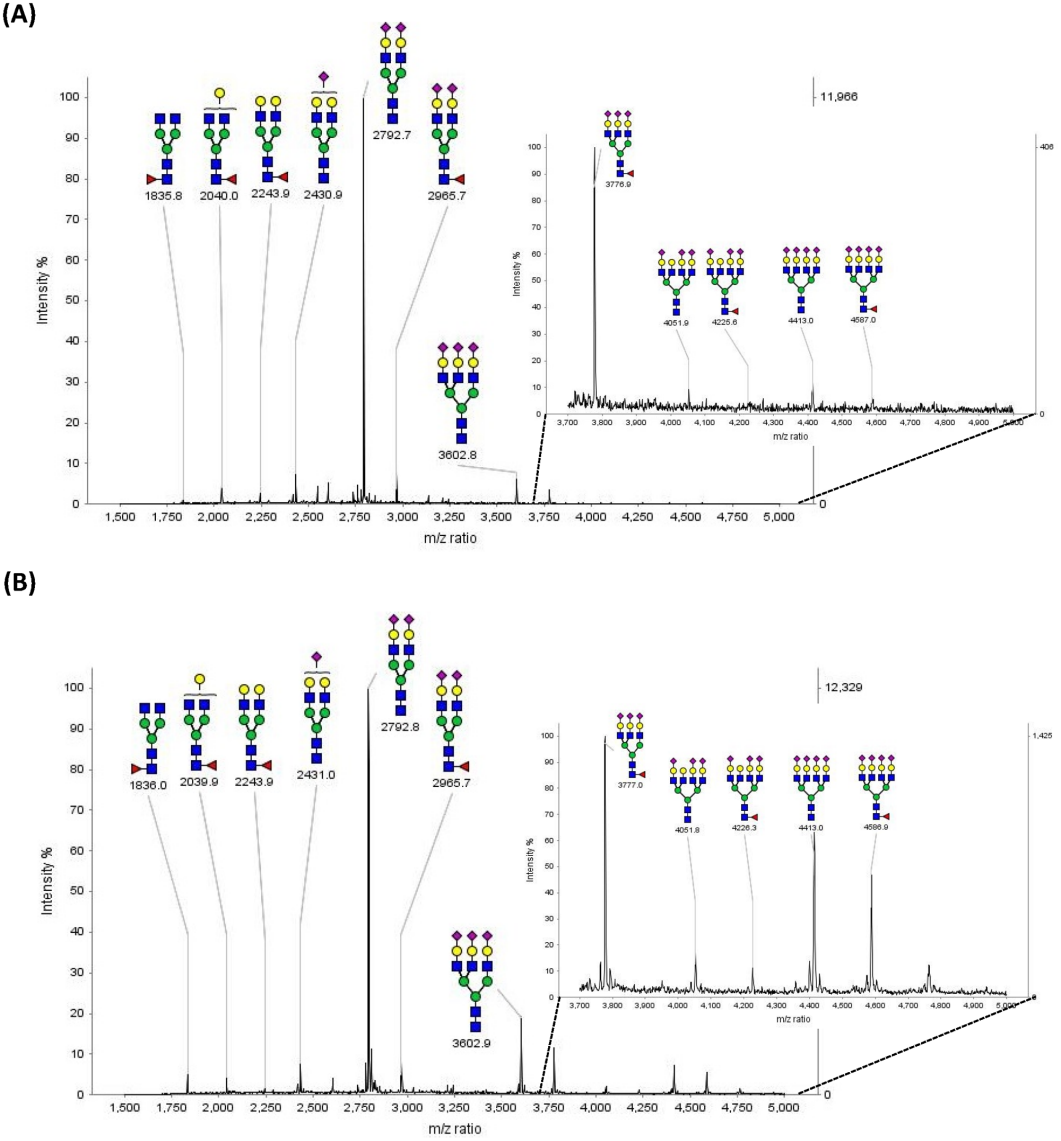
Positive ion MALDI-TOF MS spectra of permethylated *N*-glycans derived from 20 μL of (A) normal human (B) OSCC patient serum. The corresponding proposed glycan compositions and structures of the major N-glycans are shown in this spectra and listed in Table 1.

The MALDI-TOF-MS analysis of permethylated PNGase F released *N*-glycans from 20 μL serum of normal human (N = 21) (Fig 1A), and OSCC patients (N = 65) (Fig 1B) were performed. The major peaks of the spectrum and their corresponding proposed *N*-glycan compositions and structures were shown in the spectra. The MS spectrums of serum derived *N*-glycans from normal human and OSCC patients showed similar distribution patterns but a little different in the relative intensity of each glycan structure. Furthermore, by comparing these two spectrums, we found that the relative intensity of high molecular weight *N*-glycans seemed to be increased in the samples of OSCC patients. (Fig 1)

### Serum *N*-glycans which showed significantly decreased relative abundance in OSCC patient

**Fig 2:**
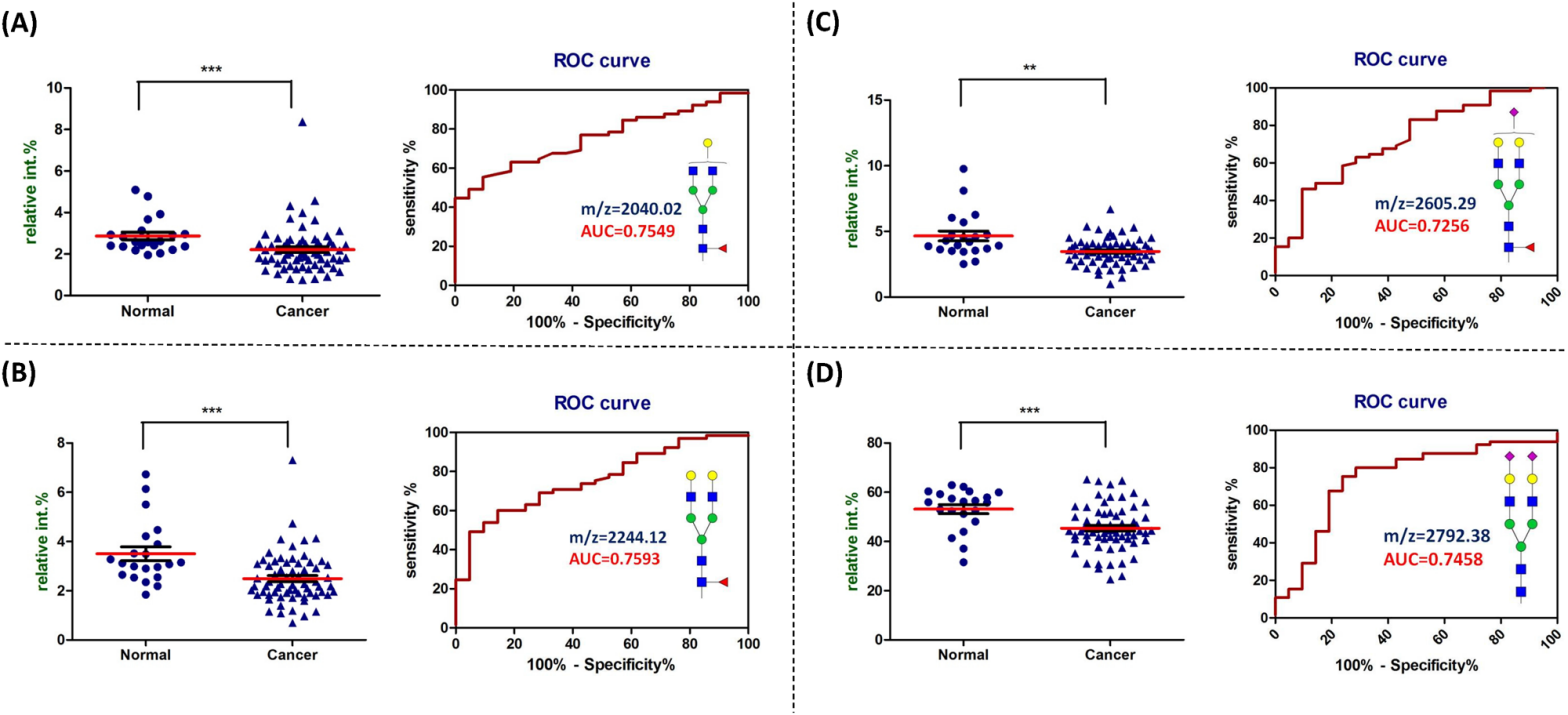
*N*-glycans which showed decreased relative abundance in cancer patient serum compared with normal volunteer. The dot plot (left) of the relative abundance and the ROC curve (right) of (**A**) monogalactosylated fucosylated bi-antennary glycan (observed at m/z = 2040.02), (**B**) fucosylated bi-antennary glycan (observed at m/z = 2244.12), (**C**) fucosylated sialylated bi-antennary glycan (observed at m/z = 2605.29), and (**D**) di-sialylated bi-antennary glycan (observed at m/z = 2792.38) in serum. The diagnostic performances are listed in S4 Table. ***, *p* < 0.001; **, *p* < 0.01 compared with normal.

As an initial step to evaluate the potential difference of serum derived *N*-glycans between normal human and OSCC patients, thirty major *N*-glycans were identified. The proposed structures of the identified *N*-glycans and their relative abundance of the total identified *N*-glycans detected by MALDI-TOF-MS in the serum were listed in detail in S3 Table. After statistical analysis on these thirty identified *N*-glycans, sixteen of these glycans showed significant difference (p value less than 0.05 and AUC value great than 0.7), and four out of sixteen exhibited decreased relative abundances in OSCC patient serum compared with normal human serum. The four *N*-glycans included monogalactosylated fucosylated bi-antennary glycan (m/z = 2040.02, Fig 2A), fucosylated bi-antennary glycan (m/z = 2244.12, Fig 2B), fucosylated sialylated bi-antennary glycan (m/z = 2605.29, Fig 2C), and di-sialylated bi-antennary glycan (m/z = 2792.38, Fig 2D). The diagnostic performance of these glycans in detecting OSCC also supported by the high AUC value, sensitivity, specificity, and accuracy showed in S4 Table.

### Serum *N*-glycans which showed significantly increased relative abundance in OSCC patient

**Fig 3:**
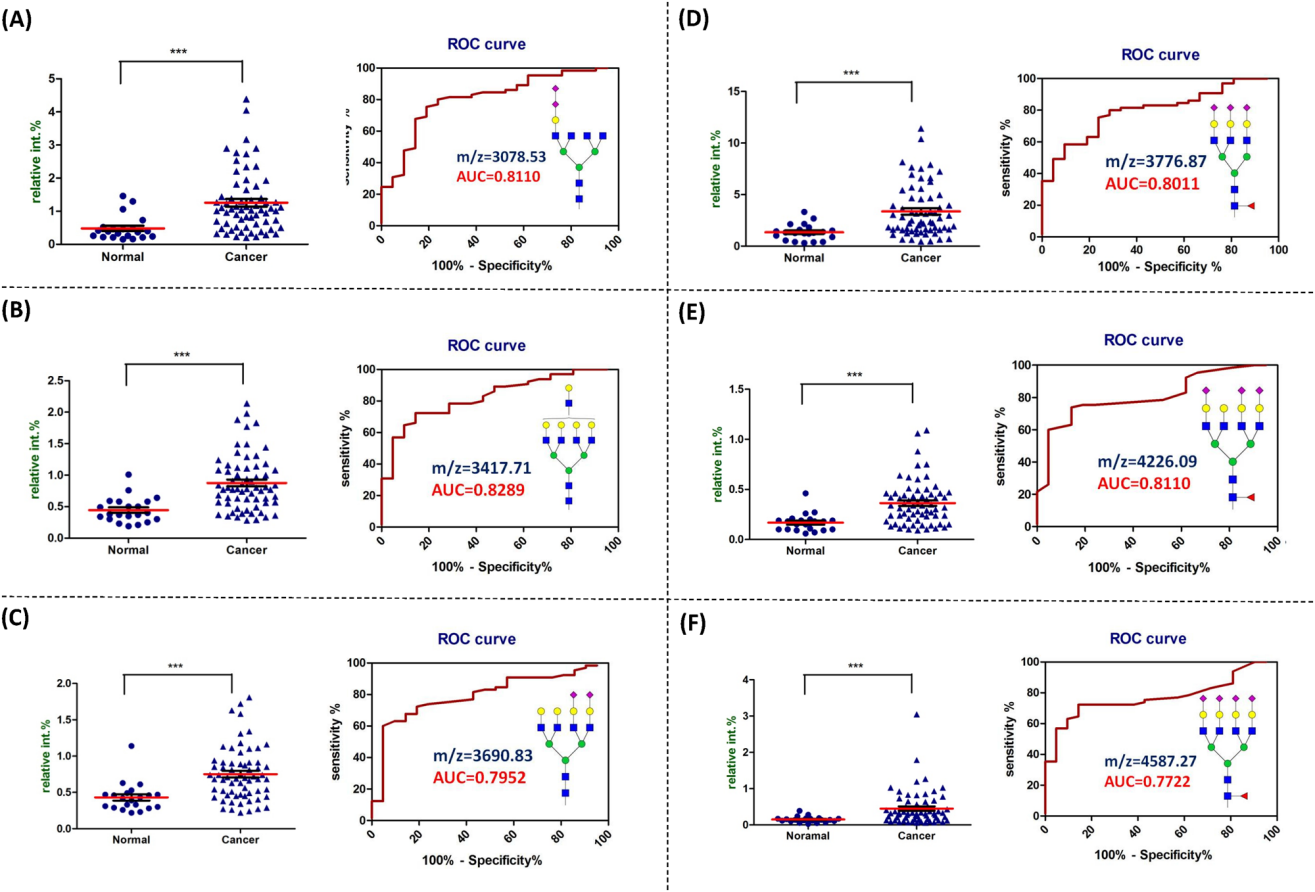
*N*-glycans which showed increased relative abundance in cancer patient serum compared with normal volunteer. The dot plot (left) of the relative abundance and the ROC curve (right) of (**A**) di-sialylated tetra-antennary glycan (observed at m/z = 3078.53), (**B**) tetra-antennary glycan (observed at m/z = 3417.71), (**C**) di-sialylated tetra-antennary glycan (observed at m/z = 3690.83), (**D**) fucosylated tri-sialylated tri-antennary glycan (observed at m/z = 3776.87), (**E**) fucosylated tri-sialylated tetra-antennary glycan (observed at m/z = 4226.09), and (**F**) fucosylated tetra-sialylated tetra-antennary glycan (observed at m/z = 4587.27) in serum. The diagnostic performances are listed in S5 Table. ***, *p* < 0.001 compared with normal.

Among the sixteen identified serum *N*-glycans which showed significant difference between normal with patient, twelve *N*-glycans showed increased relative abundances in OSCC patient serum. The dot plot of the relative abundance, ROC curve, and AUC value of *N*-glycans with AUC value higher than 0.77 were showed in Fig 3. These glycans included di-sialylated tetra-antennary glycan (m/z = 3078.53, Fig 3A), tetra-antennary glycan (m/z = 3417.71, Fig 3B), di-sialylated tetra-antennary glycan (m/z = 3690.83, Fig 3C), fucosylated tri-sialylated tri-antennary glycan (m/z = 3776.87, Fig 3D), fucosylated tri-sialylated tetra-antennary glycan (m/z = 4226.09, Fig 3E), and fucosylated tetra-sialylated tetra-antennary glycan (m/z = 4587.27, Fig 3F). The dot plot of the relative abundance, ROC curve, and AUC value of *N*-glycans with AUC value between 0.7 to 0.77 were showed in S1 Fig. These glycans included di-fucosylated bi-antennary glycan (m/z = 2418.21, S1A Fig), di-sialylated glycan (m/z = 2547.25, S1B Fig), fucosylated sialylated bisecting tetra-antennary glycan (m/z = 3136.57, S1C Fig), di-sialylated tri-antennary glycan (m/z = 3241.60, S1D Fig), tri-sialylated tetra-antennary glycan (m/z = 4052.00, S1E Fig), and fucosylated tri-sialylated tetra-antennary glycan (m/z = 4675.32, S1F Fig). The diagnostic performance of these glycans in detecting OSCC were listed in S5 and S6 Tables. Among the sixteen glycans, the diagnostic accuracy of seven glycans were greater than 75% including di-sialylated bi-antennary glycan (m/z = 2792.38), di-sialylated tetra-antennary glycan (m/z = 3078.53), di-sialylated tri-antennary glycan (m/z = 3241.60), di-sialylated tetra-antennary glycan (m/z = 3690.83), fucosylated tri-sialylated tri-antennary glycan (m/z = 3776.87), fucosylated tri-sialylated tetra-antennary glycan (m/z = 4226.09) and fucosylated tetra-sialylated tetra-antennary glycan (m/z = 4587.27).

### Serum *N*-glycan subclasses which showed significantly increased relative abundance in OSCC patient

**Fig 4:**
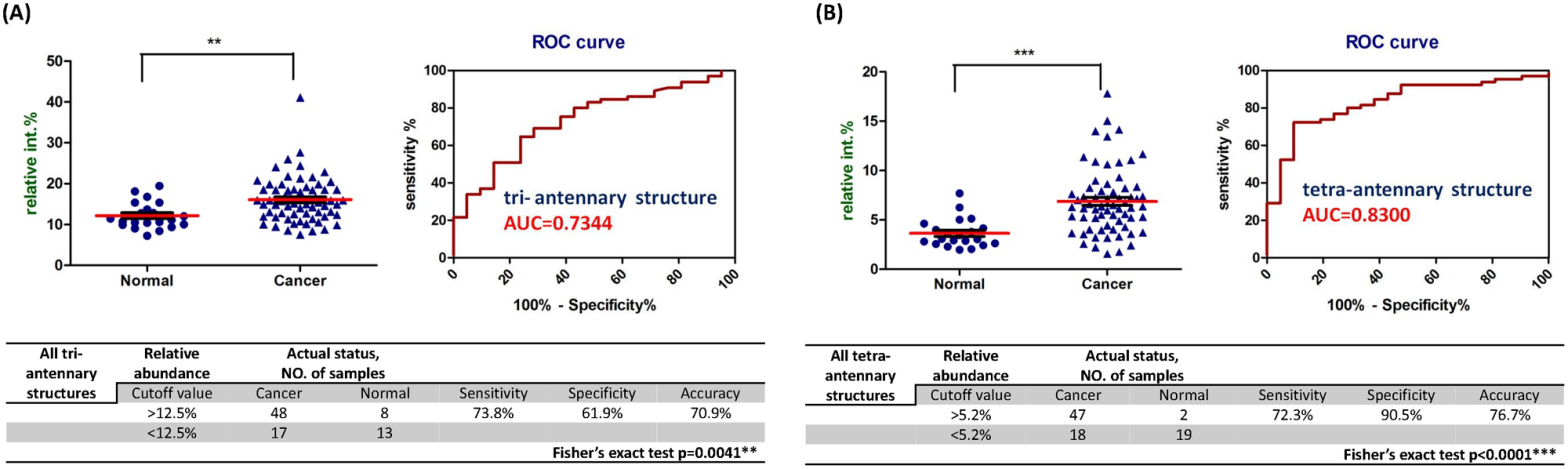
All tri-antennary and all tetra-antennary glycans showed increased relative abundance in cancer patient serum compared with normal volunteer. The dot plot (upper left) of the relative abundance, ROC curve (upper right) and the diagnostic performance (down) of the statistical results of the (**A**) all tri-antennary and (**B**) all tetra-antennary glycans in serum. ***, *p* < 0.001; **, *p* < 0.01 compared with normal.

Then, the thirty identified *N*-glycans were further divided into different subclasses including fucosylated, sialylated, tri-antennary and tetra-antennary based on their predicted characteristic structural features. The relative abundances and AUC value of each *N*-glycan subclass were listed in Table 1. We found that the relative abundances of fucosylated, tri-antennary and tetra-antennary glycans were significantly increased in the OSCC patient serums compared with normal human serums (Table 1). The diagnostic performance including dot plot of the relative abundance, ROC curve, AUC value, sensitivity, specificity, and accuracy of tri-antennary and tetra-antennary glycans were showed in Fig 4.

**Table 1:** The relative abundance of different glycan subclasses detected in normal human and OSCC patient serum.

Glycan subclass	Relative abundance(%) ± SEM		p-value	AUC
	Normal	Cancer		
	(N = 21)	(N = 65)		
**Fucosylated**	24.27 ± 1.29	27.1 ± 0.82	0.0413*	0.6491
**Sialylated**	79.27 ± 1.51	78.27 ± 0.8	0.3630	0.5667
**Tri-antennary**	12.14 ± 0.71	16.04 ± 0.68	0.0041**	0.7344
**Tetra-antennary**	3.64 ± 0.32	6.87 ± 0.42	<0.0001***	0.8300

The p-value and area-under-the-curve (AUC) are included for the comparison of normal samples and OSCC patient samples.

***, *p* < 0.001;

**, *p* < 0.01;

*, *p*<0.05, compared with normal.

### Correlation between serum *N*-glycans with the progression or lymphatic metastasis of OSCC

According to the TMN staging criteria diagnosed by clinician, 13 of the 65 OSCC patients were diagnosed in stage I of the malignancy while 16 in stage II, 16 in stage III and 19 in stage IV (S1 Table). To know whether the relative abundance of serum *N*-glycans increased or decreased with the disease progression, the relative abundance of the identified thirty *N*-glycans and different *N*-glycan subclasses in serum were statistically compared between normal human, stage I, stage II, stage III and stage IV of OSCC patients. We found that the relative abundance of fucosylated tetra-sialylated tetra-antennary glycan (m/z = 4587.27), fucosylated tri-sialylated tetra-antennary glycan (m/z = 4675.32), tri-antennary glycans, and tetra-antennary glycans showed significant differences in stages I to IV compared with normal control (S2 Fig). However, there were no significant differences between stages in the thirty identified *N*-glycans and the four *N*-glycan subclasses.

In this study, 26 of the 65 OSCC patients were diagnosed with lymphatic metastasis (in N1 or N2 stage according to the TMN staging criteria, S1 Table). In order to identify the correlation between serum *N*-glycans with lymphatic metastasis of OSCC, the relative abundance of the identified thirty *N*-glycans and different *N*-glycan subclasses in serum were statistically compared between patients with or without lymphatic metastasis. We found that the relative abundance of fucosylated di-sialylated bi-antennary glycan (m/z = 2966.47) and fucosylated tetra-sialylated tetra-antennary glycan (m/z = 4587.27) were increased in the serum sample of OSCC with lymphatic metastasis compared with other OSCC patients (S3 Fig). These glycans were potentially to be biomarkers for the diagnosis of OSCC lymphatic metastasis.

### Validation of the identified serum *N*-glycans

To verify the identified serum *N*-glycans, an independent validation group of forty-eight OSCC patients (S2 Table) and six normal volunteers was used to evaluate the sensitivity and specificity of the identified *N*-glycans (relative intensity). Based on the cutoff values we found, twelve of the eighteen identified biomarkers including m/z = 2792.38, 3078.52, 3136.57, 3241.6, 3417.71, 3690.83, 4052, 4226, 4587.27, 4675.32, tri-antennary, and tetra-antennary showed significant high sensitivity (>0.75, Table 2) and specificity (>0.83, Table 2). However, lower sensitivity (<0.75)and lower specificity (<0.7) were observed in some identified biomarkers, especially m/z = 2547.25. We also found that the relative abundance of fucosylated tetra-sialylated tetra-antennary glycan (m/z = 4587.27), fucosylated tri-sialylated tetra-antennary glycan (m/z = 4675.32) and tri-antennary glycans exhibited significant differences in stages II to IV compared with normal control in validation group (S4A–S4C Fig). In addition, the relative abundance of fucosylated di-sialylated bi-antennary glycan (m/z = 2966.47) was significantly higher in OSCC with lymphatic metastasis compared with non-metastasis OSCC patients (S4D Fig).

**Table 2:** Validation of the identified serum *N*-glycans.

m/z	Cutoff value (relative intensity)	True Positive (N = 48)	True Negative (N = 6)	Sensitivity	Specificity	References
2040.02	<2.4%	26	3	0.54	0.50	Fig 2A
2244.12	<2.95%	34	3	0.71	0.50	Fig 2B
2418.21	>1.7%	29	4	0.60	0.67	S1A Fig
2547.25	>2.65%	4	1	0.08	0.17	S1B Fig
2605.29	<3.9%	40	3	**0.83**	0.50	Fig 2C
2792.38	<52%	48	6	**1.00**	**1.00**	Fig 2D
3078.53	>0.47%	48	6	**1.00**	**1.00**	Fig 3A
3136.57	>0.7%	47	6	**0.98**	**1.00**	S1C Fig
3241.6	>1.05%	48	6	**1.00**	**1.00**	S1D Fig
3417.71	>0.5%	48	6	**1.00**	**1.00**	Fig 3B
3690.83	>0.5%	47	6	**0.98**	**1.00**	Fig 3C
3776.87	>1.5%	36	2	**0.75**	0.33	Fig 3D
4052	>0.32%	47	5	**0.98**	**0.83**	S1E Fig
4226	>0.2%	46	6	**0.96**	**1.00**	Fig 3E
4587.27	>0.19%	45	5	**0.94**	**0.83**	Fig 3F
4675.32	>0.07%	48	6	**1.00**	**1.00**	S1F Fig
Tri-antennary	>12.5%	36	6	**0.75**	**1.00**	Fig 4A
Tetra-antennary	>5.2%	47	6	**0.98**	**1.00**	Fig 4B

The serum *N*-glycan of an independent validation group of forty-eight OSCC patients and six normal volunteers were profiled, and the relative intensity of the identified *N*-glycans were analyzed. The sensitivity and specificity of the identified *N*-glycan were calculated according to the cutoff value.

### Anti-carbohydrate antibody binding profiles of normal and OSCC patient

**Fig 5:**
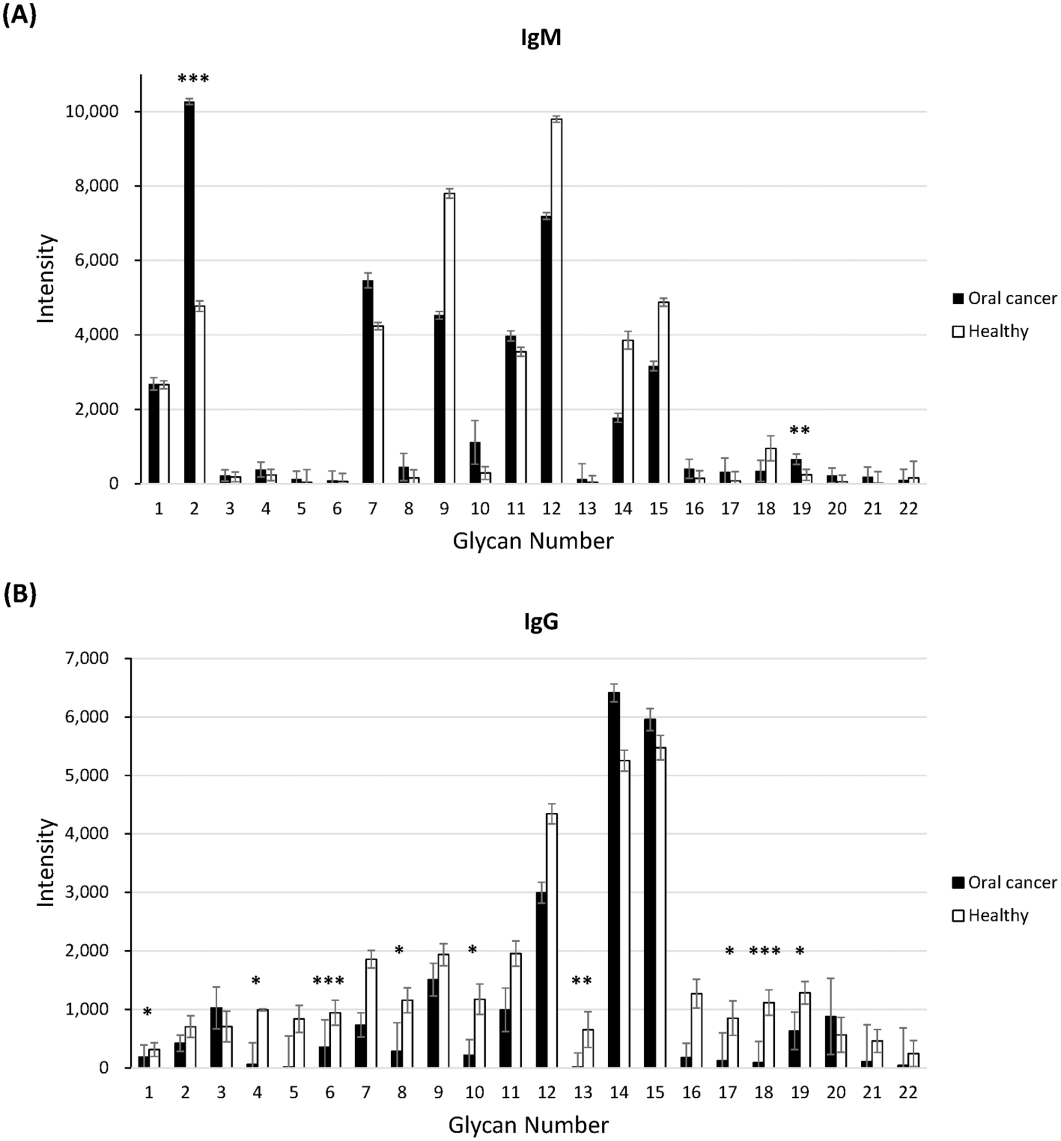
Carbohydrate binding profiles of serum (A) IgM and (B) IgG from OSCC patient and normal volunteer analyzed by Glycan-23 Chip (OBI Pharma, Inc). The data are presented as the mean ± coefficient of variation (CV) from at least three independent experiments on independent chips. ***, *p* < 0.001; **, *p* < 0.01; *, *p*<0.05, compared with normal.

Since we had identified several potential *N*-glycan biomarkers from serum of OSCC patient, we further analyzed the binding profiles of anti-carbohydrate antibodies in serum of normal and OSCC patients. An aliquot of serum was subjected into Glycan-23 chip and the binding of anti-carbohydrate antibodies (including IgG and IgM) with 22 different glycans (S7 Table) were measured. Surprisingly, we found that anti-SSEA-3 and anti-GD2 antibodies (IgM) showed significantly higher levels in OSCC patient serum than normal volunteer (Fig 5). On the contrary, the levels of nine of IgG antibodies (including anti-GHC, anti-Le^y^, anti-sialyl Le^x^, anti-NeuAc, anti-(NeuAcα2–8)_2_, anti-(NeuAcα2–8)_3_, anti-6GlcNAc-HSO_3_-sialyl Le^x^, anti-α2–6 sialylated diantennary *N*-glycan, and anti-GD2) were significantly lower in OSCC patient serum than normal volunteer (Fig 5). The intensity of selected anti-carbohydrate antibodies was listed in Table 3.

**Table 3:** The glycan binding intensity of selected anti-carbohydrate antibodies which showed statistical significance between OSCC patient with normal.

Antibody Classes	Glycan Name	Fluorescence Intensity ± CV		p-value	
		Normal	Cancer		
		(N = 21)	(N = 65)		
IgM	SSEA3	4770 ± 142	10269 ± 77	<0.001***	↑
	GD2	238 ± 147	658 ± 138	0.005**	↑
IgG	GHC	313 ± 118	187 ± 203	0.01*	↓
	Le^y^	993 ± 16	63 ± 366	0.01*	↓
	SiaLe^x^	943 ± 213	357 ± 464	<0.001***	↓
	αNeuAc-OCH_2_C_6_H_4_-p-NHCOOCH_2_	1155 ± 214	287 ± 486	0.04*	↓
	NeuAcα2-8NeuAcα (NeuAcα2–8)_2_	1172 ± 260	218 ± 265	0.01*	↓
	NeuAcα2-8NeuAcα2-8NeuAc (NeuAcα2–8)_3_	654 ± 305	16 ± 240	0.001**	↓
	Neu5Acα2-3Galβ1-4(Fucα1–3)(6-HSO_3_)GlcNAcβ (6GlcNAc-HSO_3_-SiaLe^x^)	850 ± 294	120 ± 478	0.01*	↓
	(NeuAcα2-6Gal1-4GlcNAc1-2Man)_2_α1–3,6Manα1-4GlcNAcβ1-4GlcNAc (α2–6 sialylated diantennary N-glycans)	1116 ± 216	88 ± 366	<0.001***	↓
	GD2	1284 ± 193	635 ± 319	0.02*	↓

The data are presented as the mean ± coefficient of variation (CV) from at least three independent experiments on independent chips.

***, *p* < 0.001;

**, *p* < 0.01;

*, *p*<0.05, compared with normal.

## Discussion

Several techniques have been developed for glycan analysis including the use of specific exo-glycosidases, chromatography, capillary electrophoresis, mass spectrometry, nuclear magnetic resonance and microarrays [42]. Among these methodologies, mass spectrometry is the most versatile and powerful tool for glycomic study because of its high sensitivity and low sample requirement. In addition, mass spectrometry can determine the molecular mass of analytes and elucidate the composition and structure of the glycans. The mass spectrometry can also use to identify glycoproteins and evaluate the glycosylation sites on the glycoproteins [43].

The changes in expression of *N*-linked glycan structures in the serum or tissues have been reported in many other cancers. In ovarian cancer, tri-antennary and tetra-antennary glycans with vary degrees of sialylation and fucosylation in serum are increased [12]. The same phenomenon is also found in this studies. Besides, the elevated serum fucosylation level is also observed in breast cancer [14]. However, the level of serum tri-antennary glycans is decreased in breast cancer while the level of serum tri-antennary glycans is increased in this study. Further, the decreased abundance of di-sialylated bi-antennary glycan (m/z = 2792.38) can be observed in serum of prostate cancer [13]. Nevertheless, the increased serum abundance of agalactosylated fucosylated bi-antennary glycan (m/z = 1835) and bi-antennary glycan (m/z = 2070) in prostate cancer cannot be detected in our study. The decreased relative abundance of monogalactosylated fucosylated bi-antennary glycan (m/z = 2040.02) and fucosylated bi-antennary glycan (m/z = 2244.12) can also be observed in esophageal adenocarcinoma, whereas the serum fucosylation level was decrease in esophageal adenocarcinoma [15]. Furthermore, the elevated expression level of fucosylated *N*-glycans in OSCC serum observed in this study is in consistent with previous studies which shows that the elevated fucosylation level can be found in serum and cell line of OSCC [44, 45]. Based on literature and our findings, the serum *N*-glycome of OSCC exhibited a unique profile and can be distinguished from other cancers.

Different mechanisms can account for cancer associated altered glycosylation pattern including altered expression of glycosidase, glycosyltransferase or sugar nucleotide transporters, masking of sugar epitopes by substituent groups and competition between normal and cancer-associated carbohydrate structures [46]. For example, the up-regulation of GlcNAc-transferase III (GnT III), which catalyzes the addition of "bisecting" GlcNAc, results in the increase of bisected *N*-linked glycans in ovarian cancer tissue [47]. Desiderio et al. also indicated that the increased the expression of sialyl Le^x^ (synthesized by up-regulated fucosyltransferases FUT3 and FUT6) plays important roles in the invasion of OSCC cancer stem cell [36, 48]. The findings mentioned above have pointed out several druggable targets, the glycosyltransferases, for the development of targeted therapy and precision medicine for oral cancer.

Verification of candidate serum *N*-glycan biomarkers is a necessary step in moving from the initial discovery to clinical application. To access the identified serum *N*-glycans for the diagnosis of OSCC, the serum of forty-eight OSCC patients and six normal volunteers were collected and the *N*-glycan profiles were analyzed. The significant high sensitivity and specificity of the identified *N*-glycans serves as an independent validation of and complements the current results. Some of the identified *N*-glycans also exhibited positive correlation with cancer stages or lymph node metastasis in the validation group. Although, the structures of these glycans were "predicted" by bioinformatics tool through carbohydrate database, these results are still useful information for OSCC diagnosis.

Anti-carbohydrate antibodies, A subpopulation of serum antibody, can specifically recognize normal or abnormal glycan structures [49]. Changes in the levels of anti-carbohydrate antibodies are highly associated with many diseases, pathogen infections, cancers, as well as vaccination. The levels of anti-carbohydrate antibodies are also used for clinical diagnosis of disease progression or prognosis. Since the aberrant glycan structures can be identified from cancer patient serum, serum anti-carbohydrate antibodies should be another valuable targets for cancer diagnosis [49]. For example, serum anti-TF, -Tn and α-Gal IgG levels are highly correlated with survival rate of gastrointestinal cancer [9, 50]. Plasma antibodies against sialylated and sulfated glycans including Sialyl-Tn and 6-O-Sulfo-TF are potential tumor markers for high-grade serous ovarian cancer [51]. Furthermore, serum anti-carbohydrate antibodies are also potential therapeutic molecule that against many cancers [17–20, 52]. In this study, we had applied a novel carbohydrate microarray, Glycan-23 Chip, to profile the levels of anti-carbohydrate antibodies in normal and OSCC patient serum. The Glycan-23 Chip is a microfluidic device which contains 22 different glycans including several tumor antigens (S7 Table). Serum IgG or IgM that interacts with glycans on chip was analyzed and compared between normal volunteer with cancer patient (Fig 5). Interestingly, elevated levels of the two IgM antibodies and decreased levels of nine IgG antibodies were observed. The increased level of anti-SSEA-3 may be the result of SSEA-4 overexpression in OSCC patient [53]. Besides, the decreased levels of the nine IgG antibodies (GD2, GHC, Le^y^, sialyl Le^x^, NeuAc, (NeuAcα2–8)_2_, (NeuAcα2–8)_3_, 6GlcNAc-HSO_3_-sialyl Le^x^, and α2–6 sialylated diantennary *N*-glycan) may be the consequence of antibody-antigen interaction on tumor cells [54]. According to these findings, serum anti-carbohydrate antibody profiling should be a useful approach for cancer diagnosis, as well as prognosis.

In conclusion, we applied a MS based approach to identify the potential serum *N*-glycan markers for OSCC. The increase of some tri-antennary and tetra-antennary glycans with varying degrees of fucosylation and sialylation was found in the serum of OSCC. Compared to *N*-glycan structures found in normal serum, the relative abundance of 7 *N*-glycans (observed at m/z = 2792.38, m/z = 3078.53, m/z = 3241.60, m/z = 3690.83, m/z = 3776.87, m/z = 4226.09, m/z = 4587.27) were significantly changed in the serum sample of OSCC with diagnostic accuracy greater than 75%. The change in serum abundance of glycans observed at m/z = 2966.47 and m/z = 4587.27 may be associated with the lymph node metastasis of OSCC. In addition, the results of the validation group also confirmed the possibility of clinical application of the identified serum *N*-glycans. Furthermore, the serum levels of two IgM antibodies were elevated accompanied with the decreased levels of nine IgG antibodies in patient serum. Based on our findings, it is suggested that the observed alterations in serum *N*-glycans expression and anti-carbohydrate antibody levels should be considered as potential candidates as OSCC biomarkers.

## Supporting information

S1 Fig *N*-glycans which showed increased relative abundance in cancer patient serum compared with normal volunteer. The dot plot (left) of the relative abundance and the ROC curve (right) of (**A**) di-fucosylated bi-antennary glycan (observed at m/z = 2418.21), (**B**) di-sialylated glycan (observed at m/z = 2547.25), (**C**) fucosylated sialylated bisecting tetra-antennary glycan (observed at m/z = 3136.57), (**D**) di-sialylated tri-antennary glycan (observed at m/z = 3241.60), (**E**) tri-sialylated tetra-antennary glycan (observed at m/z = 4052.00), and (**F**) fucosylated tri-sialylated tetra-antennary glycan (observed at m/z = 4675.32) in serum. The diagnostic performances are listed in S6 Table. ***, *p* < 0.001 compared with normal.
(TIFF)

S2 Fig Dot plots comparing the relative abundance of glycan structures observed in different stages of the OSCC patient serum. (**A**) fucosylated tetra-sialylated tetra-antennary glycan (observed at m/z = 4587.27), (**B**) fucosylated tri-sialylated tetra-antennary glycan (observed at m/z = 4675.32), (**C**) all tri-antennary and (**D**) all tetra-antennary glycans showed increased relative abundance accompanied with cancer stages in cancer patient serum. ***, *p* < 0.001; **, *p* < 0.01; *, *p*<0.05, compared with normal.
(TIFF)

S3 Fig Dot plots comparing the relative abundance of glycan structures observed in the serum of OSCC patients with or without lymphatic metastasis. (**A**) fucosylated di-sialylated bi-antennary glycan (observed at m/z = 2966.47) and (**B**) fucosylated tetra-sialylated tetra-antennary glycan (observed at m/z = 4587.27) showed increased relative abundance in the serum of metastatic OSCC patients compared with non-metastatic OSCC patients. ***, *p* < 0.001; **, *p* < 0.01; *, *p*<0.05, compared with normal.
(TIFF)

S4 Fig Dot plots comparing the relative abundance of glycan structures observed in different stages and metastatic OSCC patient serum (validation group). (**A**) Fucosylated tetra-sialylated tetra-antennary glycan (observed at m/z = 4587.27), (**B**) fucosylated tri-sialylated tetra-antennary glycan (observed at m/z = 4675.32), (**C**) all tri-antennary glycans showed increased relative abundance accompanied with cancer stages in cancer patient serum. (**D**) Fucosylated di-sialylated bi-antennary glycan (observed at m/z = 2966.47) showed increased relative abundance in the serum of metastatic OSCC patients compared with non-metastatic OSCC patients. ***, *p* < 0.001; **, *p* < 0.01; *, *p*<0.05, compared with normal.
(TIFF)

S1 Table The characteristics of the cancer-free volunteers and oral cancer patients (test group). (TIF)

S2 Table The characteristics of the oral cancer patients (validation group). (TIFF)

S3 Table Molecular ions and corresponding proposed *N*-glycan structures observed in the MALDI spectra of permethylated *N*-glycans from normal human and oral cancer patient serum. (PDF)

S4 Table The diagnostic performance of glycans showed in Fig 2. (TIFF)

S5 Table The diagnostic performance of glycans showed in Fig 3. (TIFF)

S6 Table The diagnostic performance of glycans showed in S1 Fig. (TIFF)

S7 Table Glycan list fabricated on Glycan-23 Chip (OBI Pharma, Inc). (TIFF)
