# Supplementary figures and images for: Serum N-glycome characterization and anti-carbohydrate antibody profiling in oral squamous cell carcinoma patients
Source: PLoS One. 2017 Jun 8;12(6):e0178927. doi: 10.1371/journal.pone.0178927 (PMC5464575; doi:10.1371/journal.pone.0178927)

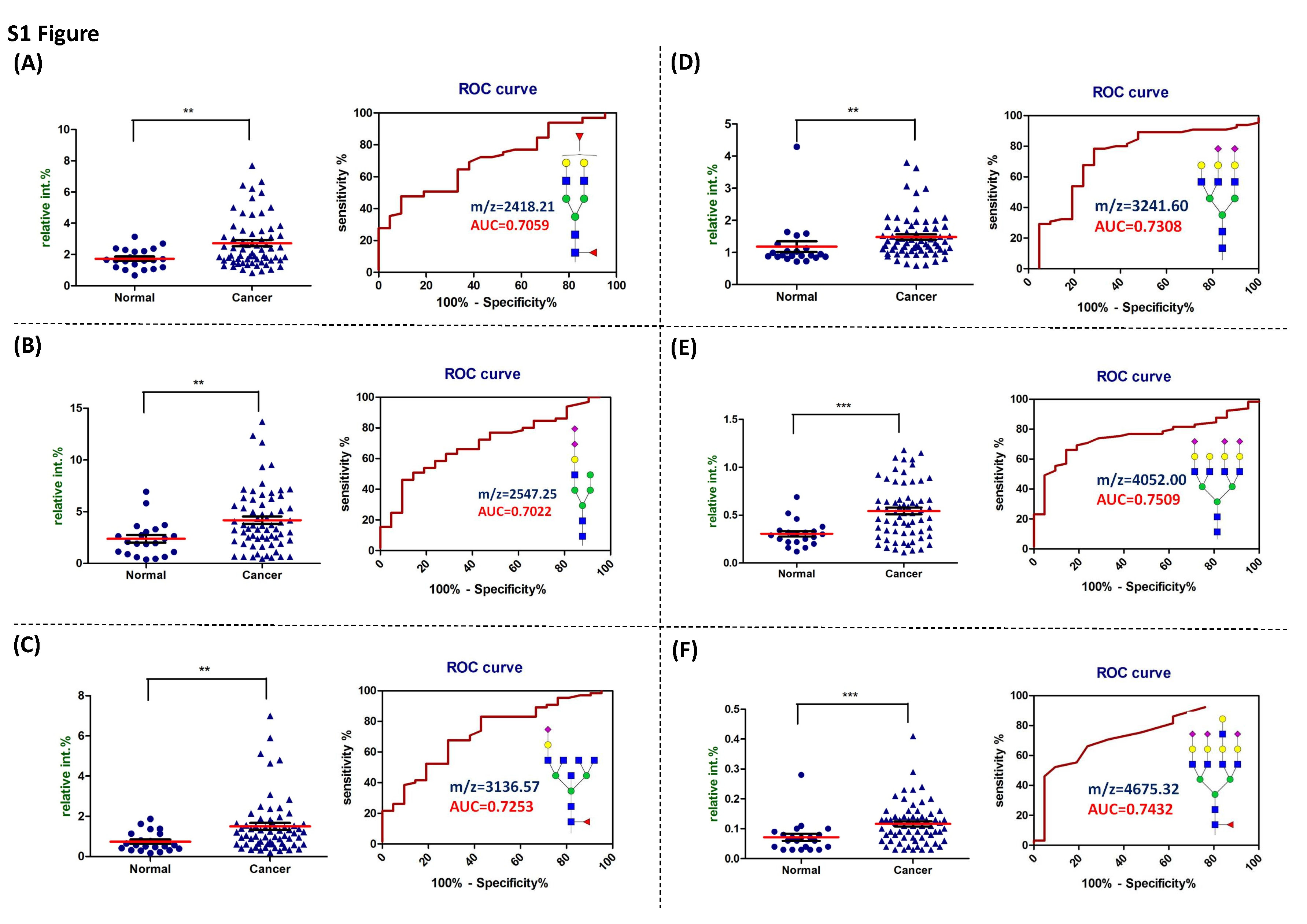

Supplement: S1 Fig — The dot plot (left) of the relative abundance and the ROC curve (right) of (A) di-fucosylated bi-antennary glycan (observed at m/z = 2418.21), (B) di-sialylated glycan (observed at m/z = 2547.25), (C) fucosylated sialylated bisecting tetra-antennary glycan (observed at m/z = 3136.57), (D) di-sialylated tri-antennary glycan (observed at m/z = 3241.60), (E) tri-sialylated tetra-antennary glycan (observed at m/z = 4052.00), and (F) fucosylated tri-sialylated tetra-antennary glycan (observed at m/z = 4675.32) in serum. The diagnostic performances are listed in S6 Table. ***, p < 0.001 compared with normal. (TIFF) [file pone.0178927.s001.tiff]

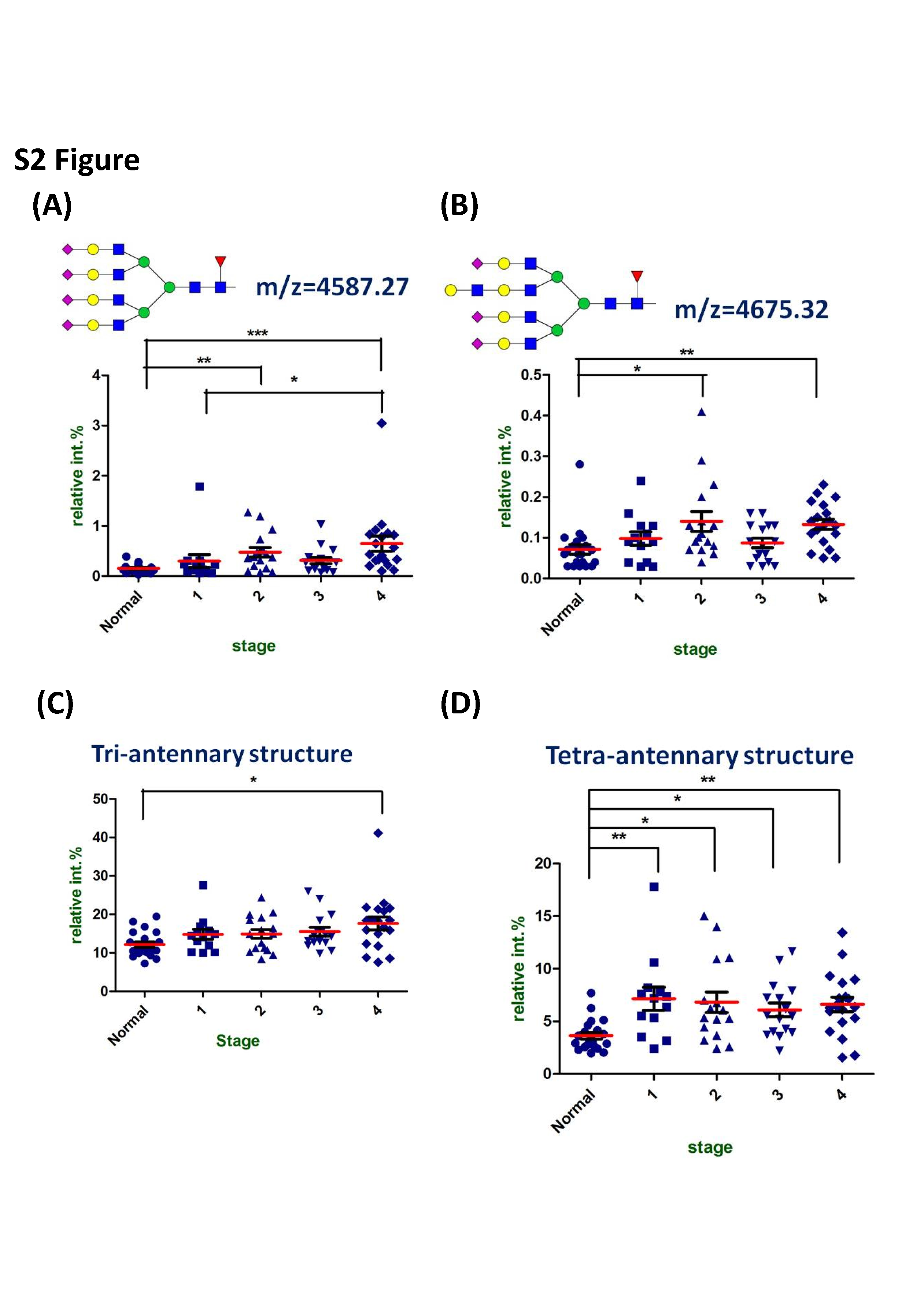

Supplement: S2 Fig — (A) fucosylated tetra-sialylated tetra-antennary glycan (observed at m/z = 4587.27), (B) fucosylated tri-sialylated tetra-antennary glycan (observed at m/z = 4675.32), (C) all tri-antennary and (D) all tetra-antennary glycans showed increased relative abundance accompanied with cancer stages in cancer patient serum. ***, p < 0.001; **, p < 0.01; *, p<0.05, compared with normal. (TIFF) [file pone.0178927.s002.tiff]

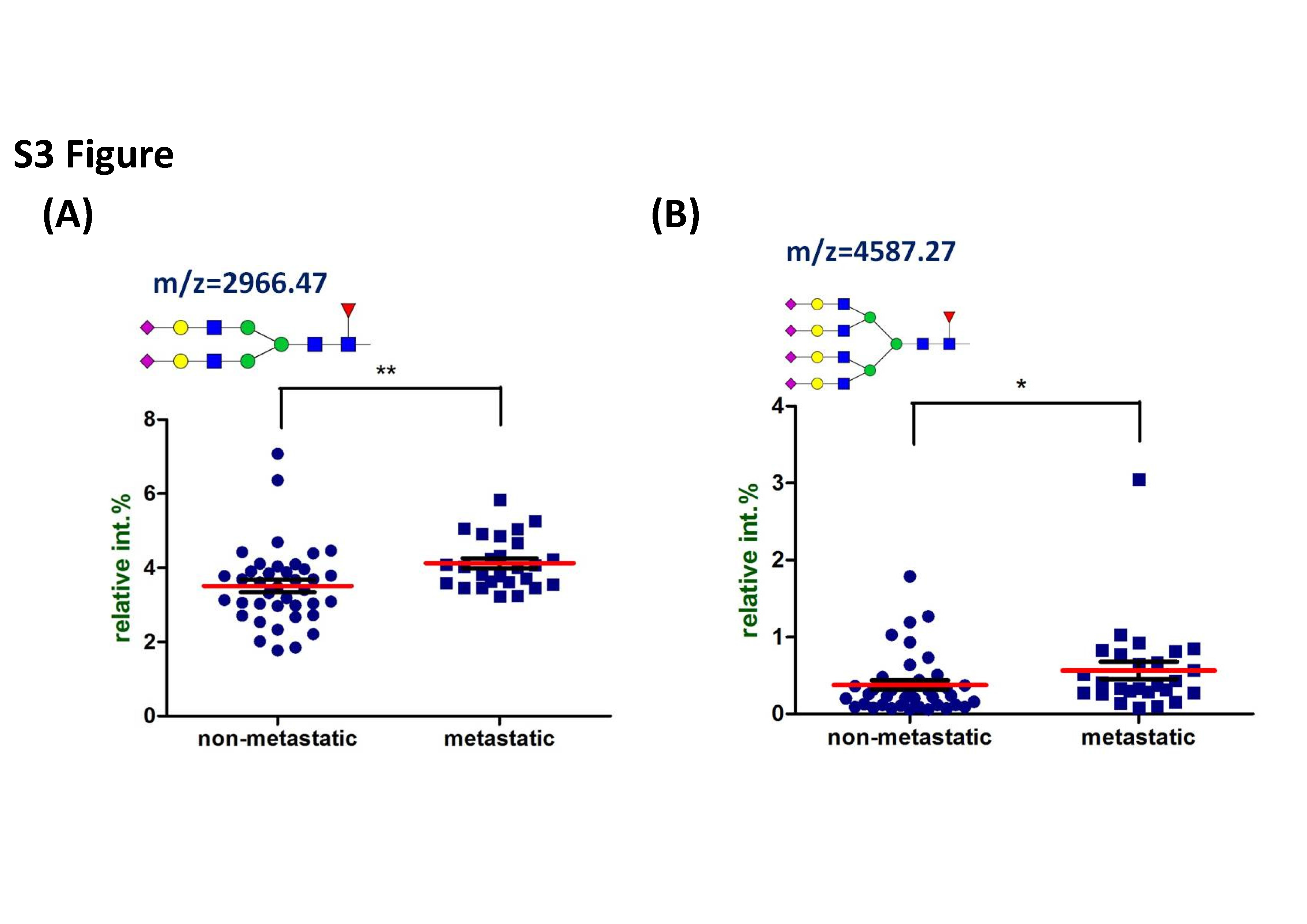

Supplement: S3 Fig — (A) fucosylated di-sialylated bi-antennary glycan (observed at m/z = 2966.47) and (B) fucosylated tetra-sialylated tetra-antennary glycan (observed at m/z = 4587.27) showed increased relative abundance in the serum of metastatic OSCC patients compared with non-metastatic OSCC patients. ***, p < 0.001; **, p < 0.01; *, p<0.05, compared with normal. (TIFF) [file pone.0178927.s003.tiff]

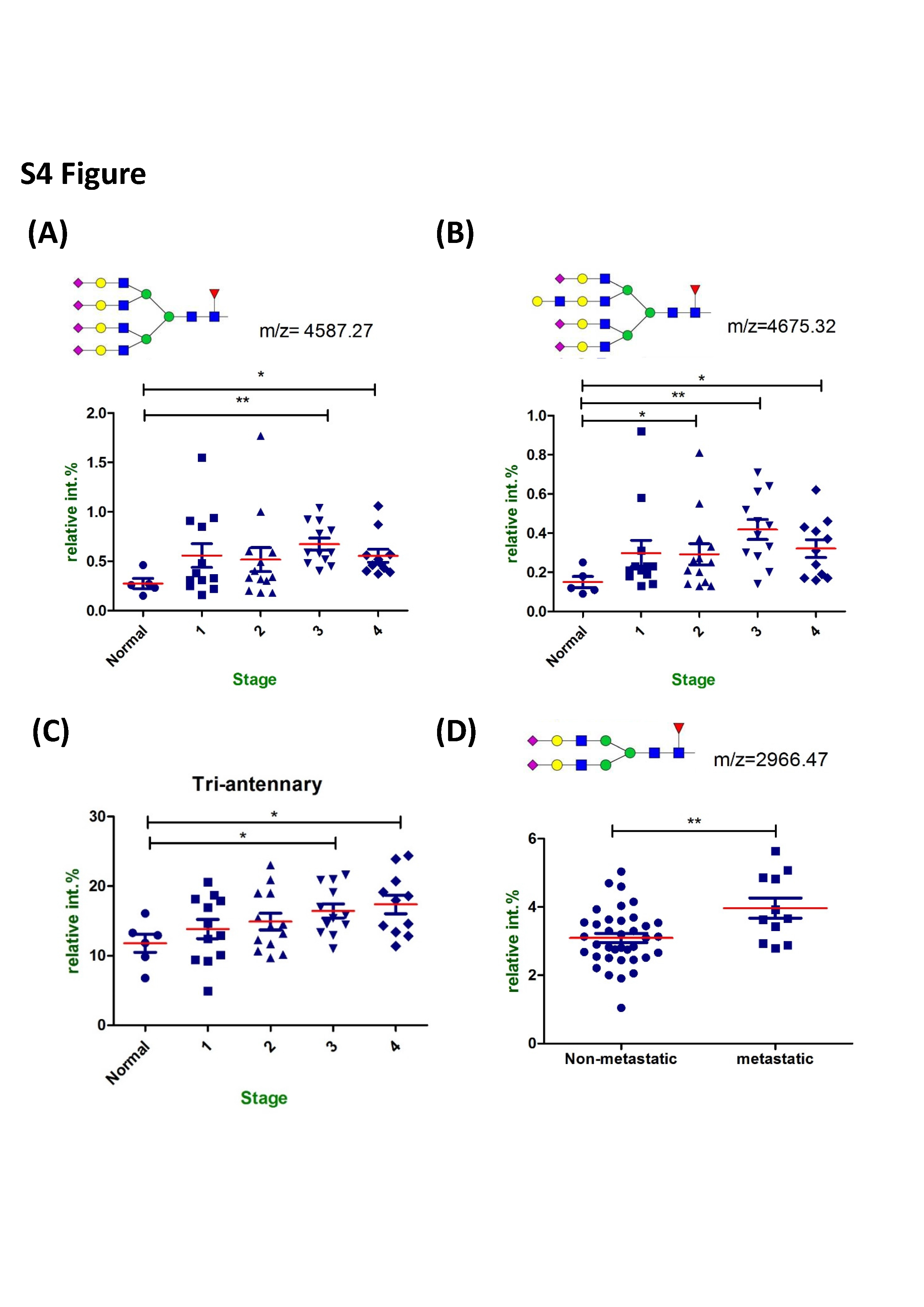

Supplement: S4 Fig — (A) Fucosylated tetra-sialylated tetra-antennary glycan (observed at m/z = 4587.27), (B) fucosylated tri-sialylated tetra-antennary glycan (observed at m/z = 4675.32), (C) all tri-antennary glycans showed increased relative abundance accompanied with cancer stages in cancer patient serum. (D) Fucosylated di-sialylated bi-antennary glycan (observed at m/z = 2966.47) showed increased relative abundance in the serum of metastatic OSCC patients compared with non-metastatic OSCC patients. ***, p < 0.001; **, p < 0.01; *, p<0.05, compared with normal. (TIFF) [file pone.0178927.s004.tiff]

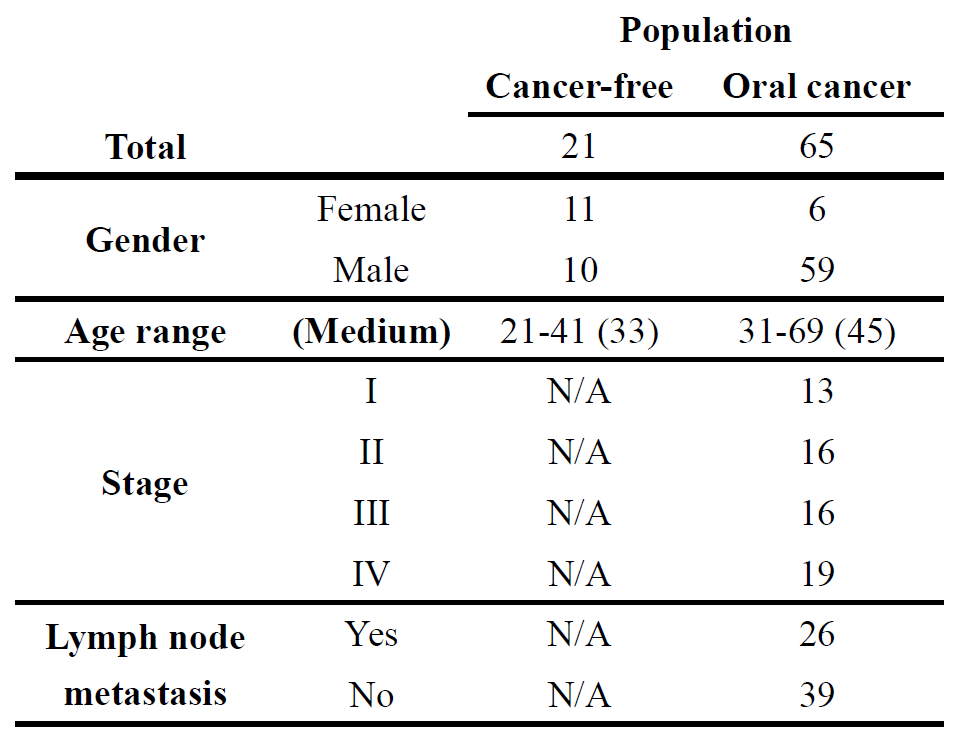

Supplement: S1 Table — (TIF) [file pone.0178927.s005.tif]

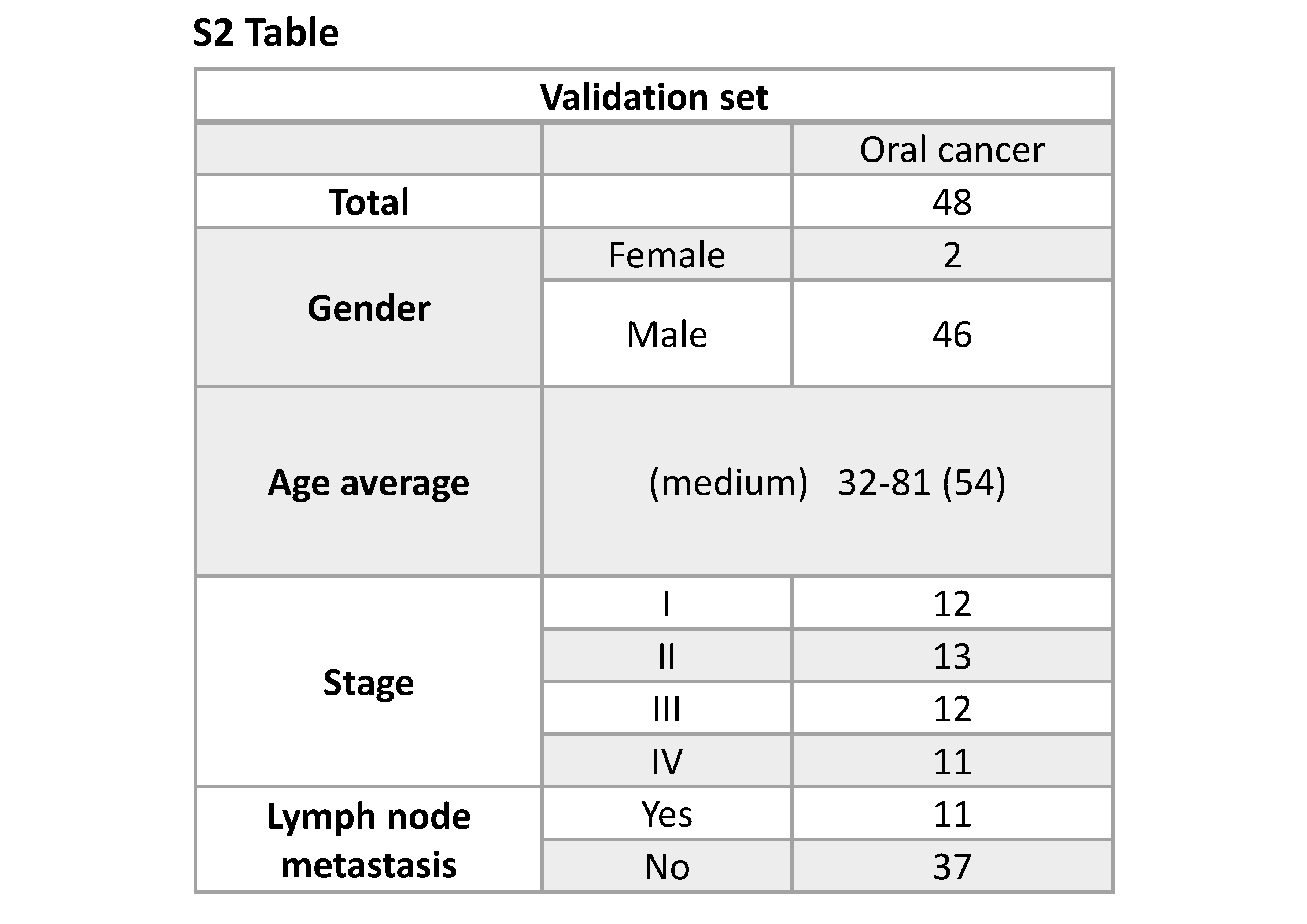

Supplement: S2 Table — (TIFF) [file pone.0178927.s006.tiff]
