# Supplementary material for: Serum N-glycome characterization and anti-carbohydrate antibody profiling in oral squamous cell carcinoma patients
Source: PLoS One. 2017 Jun 8;12(6):e0178927. doi: 10.1371/journal.pone.0178927 (PMC5464575; doi:10.1371/journal.pone.0178927)
Supplement: S3 Table — (PDF) [file pone.0178927.s007.pdf]

S3 Table. Molecular ions and corresponding proposed *N*-glycan structures observed in the MALDI spectra of permethylated *N*-glycans from normal human and oral cancer patient serum.

Relative abundance of each proposed *N*-glycan structure is calculated by expressing the intensity of each glycan ion as percent of the total intensity of all glycans. The p-value and area-under-the-curve(AUC) are included for the comparison of normal samples and oral cancer patient samples.

*SEM: standard error of the mean

m/z [M+Na$^+$]	Proposed structure	Relative abundance (%) ±SEM		p-value	AUC
		Normal (N=21)	Cancer (N=65)		
1579.78	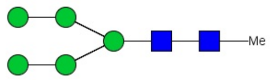	0.08 ± 0.02	0.1 ± 0.01	0.0838	0.6260
1620.80	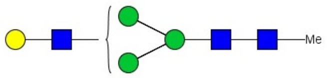	0.05 ± 0.01	0.06 ± 0.01	0.4423	0.5557
1783.88	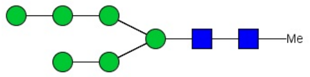	0.3 ± 0.04	0.34 ± 0.03	0.5419	0.5663
1835.92	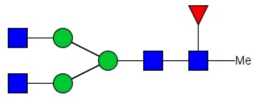	0.92 ± 0.1	1.08 ± 0.07	0.1700	0.6004
1981.98	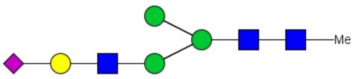	0.45 ± 0.08	0.53 ± 0.04	0.1381	0.6084
2040.02	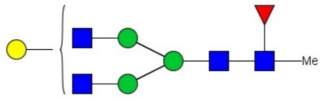	2.87 ± 0.18	2.21 ± 0.14	0.0005***	0.7549
2070.03	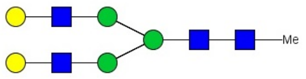	2.17 ± 0.09	2.07 ± 0.05	0.3901	0.5630

S3 Table. Continued

m/z [M+Na⁺]	Proposed structure	Relative abundance (%) ±SEM		p-value	AUC
		Normal (N=21)	Cancer (N=65)		
2227.10	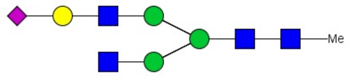	0.69 ± 0.08	0.83 ± 0.04	0.0594	0.6377
2244.12	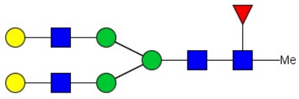	3.5 ± 0.28	2.49 ± 0.13	0.0004***	0.7593
2418.21	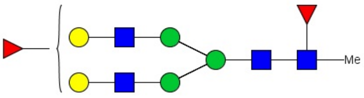	1.73 ± 0.14	2.72 ± 0.2	0.0048**	0.7059
2431.20	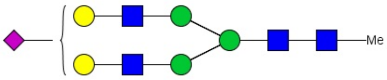	8.02 ± 1.27	8.15 ± 0.59	0.4271	0.5582
2459.24	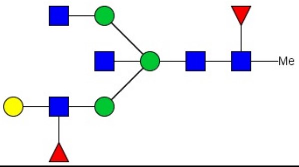	0.68 ± 0.09	0.87 ± 0.05	0.0210*	0.6685
2547.25	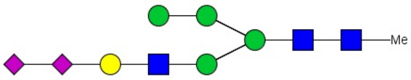	2.38 ± 0.37	4.19 ± 0.36	0.0056**	0.7022
2605.29	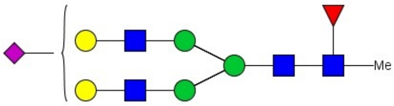	4.66 ± 0.38	3.47 ± 0.12	0.0020**	0.7256
2738.36	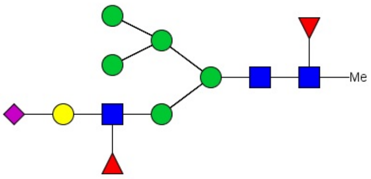	2.12 ± 0.13	2.52 ± 0.13	0.0894	0.6242
2792.38	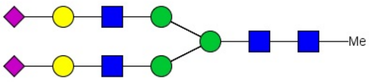	53.16 ± 1.84	45.38 ± 1.15	0.0008***	0.7458

S3 Table. Continued

m/z [M+Na$^+$]	Proposed structure	Relative abundance (%) ±SEM		p-value	AUC
		Normal (N=21)	Cancer (N=65)		
2826.41	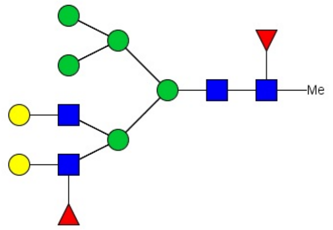	0.99 ± 0.11	1.3 ± 0.09	0.0488**	0.6440
2966.47	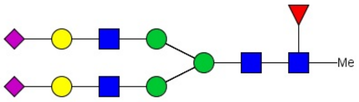	3.89 ± 0.25	3.76 ± 0.12	0.9719	0.5029
3078.53	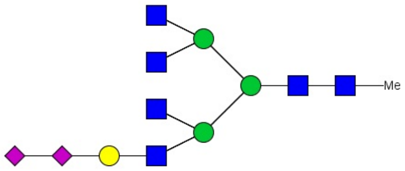	0.48 ± 0.08	1.23 ± 0.11	<0.0001***	0.8110
3136.57	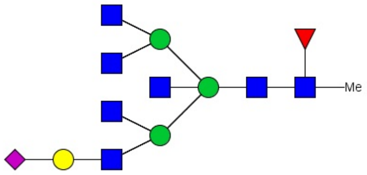	0.74 ± 0.11	1.5 ± 0.17	0.0020**	0.7253
3241.60	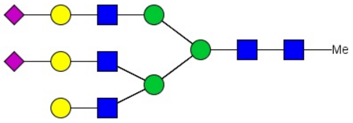	1.18 ± 0.17	1.48 ± 0.08	0.0016**	0.7308
3417.71	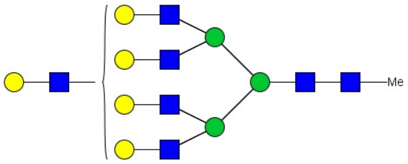	0.44 ± 0.04	0.88 ± 0.05	<0.0001***	0.8289

S3 Table. Continued

m/z [M+Na^{+}]	Proposed structure	Relative abundance (%) ±SEM		p-value	AUC
		Normal (N=21)	Cancer (N=65)		
3602.78	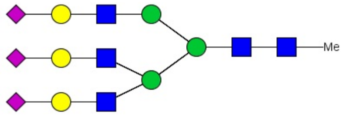	7.05 ± 0.72	7.8 ± 0.61	0.5167	0.5476
3690.83	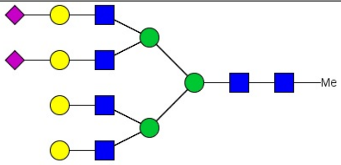	0.43 ± 0.34	0.75 ± 0.66	<0.0001***	0.7952
3776.87	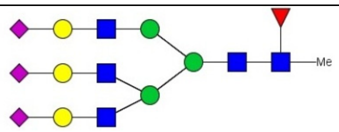	1.35 ± 0.17	3.36 ± 0.31	<0.0001***	0.8011
4052.00	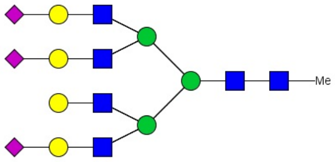	0.31 ± 0.03	0.54 ± 0.04	0.0006***	0.7509
4226.09	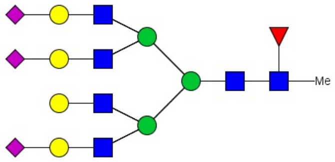	0.17 ± 0.02	0.36 ± 0.03	<0.0001***	0.8110
4413.18	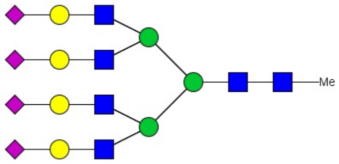	0.31 ± 0.04	0.59 ± 0.07	0.0314*	0.6571
4587.27	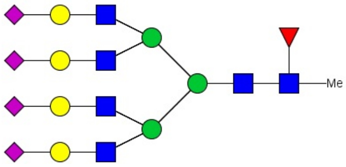	0.15 ± 0.02	0.45 ± 0.06	0.0002***	0.7722
4675.32	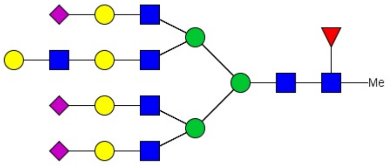	0.07 ± 0.01	0.12 ± 0.01	0.0008***	0.7432
